# Preparation and Characterization of Thermal Storage Ceramics from Iron-Containing Solid Waste

**DOI:** 10.3390/ma18040909

**Published:** 2025-02-19

**Authors:** Cheng Xue, Peiyang Lu, Zhiwei Wu, Yu Li

**Affiliations:** State Key Laboratory of Advanced Metallurgy, School of Metallurgical and Ecological Engineering, University of Science and Technology Beijing, Beijing 100083, China; xctengfeiwls@gmail.com (C.X.);

**Keywords:** solid waste, thermal storage, ceramic material, iron-containing

## Abstract

Copper slag and red mud with high iron contents were discharged with an annual global amount of 37.7 and 175 million tons but had low utilization rates due to wide reuse difficulties. Studies on their large-scale utilization have become urgent. Thermal storage ceramic is a kind of energy storage material with high-added value and a potentially large market. In this study, a method to convert copper slag and red mud into thermal storage ceramics through a ceramic fabrication process was proposed. Four samples were prepared and characterized by XRD and SEM-EDS, as well as physical and thermal property tests. The relationships among phase composition, microstructure, and properties were further discussed. The results showed the thermal storage ceramic from copper slag had the best properties with a flexural strength of 68.02 MPa and a thermal storage density of 1238.25 J/g, both equal and nearly twice those of traditional heat storage materials like Magnesia Fire Bricks and corundum. The grain sizes of mineral phases in the prepared thermal storage ceramics have significant impacts on the performance of the material. Increasing the proportion of copper slag in thermal storage ceramics from red mud could enhance their performance. This study provides a new perspective on the low-cost preparation of thermal storage ceramics and large-scale utilization of iron-containing solid waste.

## 1. Introduction

Both aluminum and copper industries are important to the non-ferrous metal industry, and their prosperity contributes to the progress of society. Meanwhile, problems of solid waste treatment occurred. Copper slag is a kind of solid waste produced from copper separation, which contains unused metals like Fe, Cu, Al, Zn, and Ni. Red mud is the industrial solid waste residue from the aluminum and alkali industry. The global annual production of copper slag amounts to 37.7 million tons [1], and red mud has an estimated global stockpile of over 4 billion tons [2] and an annual production of approximately 175 million tons [3]. Their high discharge levels and low utilization rate lead to wastes of valuable resources, lands being occupied, and soil and water pollution due to the high alkalinity, heavy metal, and radioactive properties. Hence, it has become an urgent task to achieve the high value-added resource utilization of copper slag and red mud [4,5]. Many researchers have proposed various ways to manage copper slag, such as metal recovery [6,7,8], building material preparation like ceramics and cement, and production of value-added products [9,10]. Also, considerable advancements have been made in the application of red mud such as building material production and waste substances treatment [11,12,13]. The addition of red mud could enhance materials’ certain properties like durability and strength, and it is estimated that around 600,000 to 1,800,000 tons of red mud were recycled in construction material production, such as bricks, cement, ceramics and tiles [14]. Some researchers also studied the heavy metal leaching performances of such materials for further applications [15,16,17].

In recent years, the problem of energy shortage has raised great concern. The time inconsistency of energy demand and supply urgently calls for efficient energy storage systems. Compared with energy storage systems like hydrogen and electrochemical energy storage systems, thermal storage typically has lower technical requirements. Thermal storage ceramics (TSCs) are core components of thermal storage systems and there is a pressing need to make breakthroughs in terms of TSCs’ properties like specific heat capacity and thermal conductivity with especially low product cost [18,19]. Thermal storage materials could be categorized into thermochemical heat storage material, phase change material and sensible heat storage material, which is the one this study mainly focused on. Compared with phase change materials, sensible heat storage materials exhibit simplicity in design and implementation, along with cost-effectiveness and chemical stability. However, they have lower thermal storage density typically, requiring larger volumes and their operational temperature range is limited. Compared with thermochemical heat storage materials, sensible heat storage materials are low-cost, safer and more stable, but the temperature range is less flexible, and storage density is much lower [20,21,22,23,24].

Using industrial solid waste to prepare thermal storage materials could not only achieve the utilization of solid waste but also promote the decreased cost and wide application of TSCs.

Currently, several types of research have focused on the preparation of thermal storage materials from various kinds of industrial solid waste, such as steel slag, bauxite tailing, fly ash and so on. Jingcen Zhang et al. [25] prepared a novel thermal storage material with good mechanical and thermal properties from low-cost steel slag combined with magnesium oxide and clay, and the main crystalline phases are periclase and magnetite. Bo Shi et al. [26] prepared a new type of composite heat storage material using steel slag as the matrix, MgO as an additive, and clay as the binder with a heat capacity at 720 °C reaching 1.27 J/(g·K). Xiaohong Xu et al. [27] used high calcium and high iron steel slag as main materials by adding cordierite to prepare solar thermal storage ceramics with heat capacity reaching 1.36 J/(g·K). Daniel Bielsa et al. [28] studied the use of steel slag as an energy storage material and air as a heat transfer medium and made a testing prototype, further proving the viability of using steel slag as a heat storage material. Hmida Slimani et al. [29] used an electric arc furnace (EAF) slag and prepared thermal storage material successfully. Michael Krüger et al. [30] applied sintered EAF steel slags into solid heat storage systems in a solar tower power plant. Kholoud M. Al Naimi et al. [31] utilized EAF steel slags for the preparation of potential storage media for thermal energy storage systems. Yasir Saleem et al. [32] proposed a heat storage tank composed of concrete and heat transfer fluid passages. Jianfeng Wu et al. [33] studied the effect of CaO/SiO_2_ ratio and temperature on the ceramic properties by the preparation of thermal energy storage ceramic from magnesium slag. Thus, utilizing low-cost industrial solid waste as raw materials to prepare TSCs has viability.

The past decades have witnessed great progress in the improvement of TSCs’ thermal properties via effective strategies such as doping and additives, structural optimization, Encapsulation and so on. NematpourKeshteli Abolfazl et al. [34] proposed a method of employing metal foam, nanoparticles and fins to reduce the melting time of phase change TSCs and improve the thermal properties accordingly. Attia Mohammed El Hadi et al. [35] improved the accumulative thermal efficiency via the optimization of marble constructions. Msmali Ahmed H. et al. [36] used nanoparticles with various sizes to achieve the best design of thermal storage units. NematpourKeshteli Abolfazl et al. [37] proposed a lobed double-pipe heat exchanger with a solar parabolic system that can store and release energy, and the charging and discharging rate of the thermal storage system was increased. Li Min et al. [38] used paraffine and graphene oxide to prepare a encapsulate which could improve the thermal properties of the thermal materials. Natalia Ra’zny et al. [39] produced honey-comb-shaped structures by 3D printing to improve heat transfer properties. Gunjo D. G. et al. [40] proposed a method of using a paraffin-based nanofluid blended with aluminum oxide nanoparticles as phase change materials that could improve the thermal performance of the system. Anagnostopoulos Argyrios et al. [2] used red mud and molten salt to prepare the composite material, which is ideal for waste heat recovery.

Moreover, different research indicated that the thermal properties of TSCs could be improved by adding high ferrite additives. Ying Yang et al. [41] produced the red mud/Mn co-doped high alumina cement-stabilized carbide slag for a heat storage system and found that the existence of red mud and Mn could improve the basicity and heat storage rate of the carbide slag. Mingjun Rao et al. [42] utilized laterite ore tailing to prepare heat storage material, and the specific heat capacity could reach 0.79 J/(g·K). Zongce Chai et al. [43] studied the addition of magnetite on the performances of thermal storage ceramics prepared from the bauxite tailings and red mud, the optimal sample’s specific heat capacity of 1.48 J/(g·K) and mineral phases of hematite and corundum. Xiao-hon Xu et al. [44] prepared thermal storage ceramics from red mud and shale, and the main phase compositions were hematite and kyanite. Qi Wang et al. [45] proposed a method for the preparation of ferric-rich ceramics with high storage capacity by the addition of Fe_2_O_3_ powder to bauxite tailings and found the crystalline phase transformed into corundum, hematite and mullite, which have high heat capacity properties. Xiaohong Xu et al. [46] prepared a kind of corundum thermal storage ceramics doped with Fe_2_O_3_ and TiO_2_ with its specific heat capacity of 1.22 J/(g·K) and thermal conductivity of 5.96 W/(m·K) at 800 °C. Jiangfeng Li et al. [47] utilized red mud and fly ash to prepare TSCs and found the addition of red mud could promote liquid phase formation, which improved thermal storage properties. To provide a clearer understanding for readers. Table 1 presents the details of the materials discussed in the aforementioned literature.

Both copper slag and red mud are iron-rich bulk solid wastes, with iron oxide contents of 42.83% and 67.32%, respectively. The above researches indicate that the preparation of TSCs using iron-containing industrial solid waste has viability. Thus, utilizing them for TSC preparation could be potential. In addition, the crystalline phases and the iron contents of the material have great influences on thermal storage performances. Therefore, TSCs with good performances could be obtained in appropriate conditions. Compared to current research, this study proposed a thermal storage material preparation method of using iron-containing solid waste specifically and how grain sizes of the mineral phases could exert an impact on materials’ thermal performances.

In this study, a method was proposed to prepare TSCs by using high iron content solid waste. As typical iron-containing solid wastes, red mud and copper slag were utilized to prepare different thermal storage materials in the procedure of crush and milling, granulation and compaction, drying and sintering. Materials were analyzed by XRD (X-ray diffraction analysis), SEM-EDS (Scanning Electron Microscopy-Energy Dispersive Spectrometer) and TG-DSC (Thermogravimetric-Differential Scanning Calorimetry) analysis with mechanical and thermal properties tested. Therefore, how the mineral phases and microstructure affect TSCs’ properties was discussed accordingly. This study provides new research ideas for the high-value-added utilization of bulk solid waste and the low-cost preparation of thermal storage materials.

## 2. Materials and Methods

### 2.1. Raw Materials

Copper slag and red mud are raw materials of the TSCs. Copper slag is from CHINA COPPER Co., Kunming, China. and is categorized into high iron copper slag (HCS) and copper slag (CS) according to the iron contents, while HCS is from the magnetic separation process of CS. Red mud (RM) is from CHALCO., Zibo, China. Their chemical compositions are shown in Table 2. Both CS, HCS and RM were crushed and sieved. Powder with particle size < 187.5 mm was selected for subsequent works. As shown in Table 2, the iron oxide content of RM, HCS and CS is 67.32%, 66.79% and 42.83%, respectively. HCS and RM have close iron oxide contents. The content of LOI (Loss On Ignition) was listed, which is related to volatile matter like crystal water, bound water, sulfide, carbonate and so on. “Others” except LOI in the table includes the substances with relatively low contents. Most of their contents are below 0.1%, and may not be that precise according to XRF tests and may need chemical or other ways to measure precisely.

### 2.2. Sample Preparation

The 3 kinds of raw materials were fully mixed in different mass ratios as shown in Table 3, and approximately 1–2% of water was added to ensure the stability. Then, the mixtures were pressed into a cuboid block of 50 mm × 7 mm × 6 mm under 30 MPa for 30 s and then dried at 105 °C in the drying oven (Model DHG-9140A, Shanghai Yiheng Technology Instrument Co., Shanghai, China) for over 10 h. Then, the block mixtures were sintered at 1100 °C, 1120 °C, 1140 °C, 1160 °C, 1180 °C and 1200 °C in the gradient furnace (Model GR1300/13, Nabertherm, Breman, Germany) with the heating rate of 5 °C per minute and soaking time of 1.5 h in an air atmosphere. Then, samples were grouped and numbered. C-RM were sampled from 100% RM, C-HCS were sampled from total HCS, C-CS were made from CS, and C-MIX were from the mixture of 80% HCS and 20% RM. It is worth noting that C-MIX was meant to modify the content of Al and Si and meanwhile keep the content of Fe high. Then, different sintering temperatures were labeled from 1 to 6. For example, the sample from C-MIX sintered at 1120 °C was named C-MIX2.

### 2.3. Characterization

The chemical composition of raw materials was measured using a Wavelength Dispersive X-ray Fluorescence Spectrometer (WDXRF; ZSX Primus III+, Rigaku Ultima, Rigaku, Osaka, Japan; Testing voltage of 50 kV; Testing current of 60 mA). Samples used in the test were powder with particle size < 187.5 μm.

The phase composition of the sample was determined by X-ray diffraction analysis (XRD; Rigaku Ultima, Rigaku, Tokyo, Japan; Testing voltage of 40 kV; Testing current of 40 mA; Testing wavelength of 0.15418 nm). The scanning angle (2θ) is 10–90°. The sample used in this test was powder with a particle size < 187.5 μm.

The microstructure of the sample was speculated by SEM-EDS (Scanning Electron Microscopy-Energy Dispersive Spectrometer; GeminiSEM 300, Carl Zeiss AG, Oberkochen, Germany), and samples used in this test were cuboid blocks embedded in resin with gold coating.

The flexural strength of the sample was tested on a three-point bending strength tester (Model BLR-12, Changsha, China), and the flexural strength was obtained by Formula (1) [48]. Water absorption is obtained according to Formula (2) [49]. Samples used were cuboid blocks of 50 mm × 7 mm × 6 mm.

Specific heat capacity (C_p_) was tested via calorimetric method by TG-DSC (Thermogravimetric-Differential Scanning Calorimetry; Model HCT, Beijing Hengjiu Co., Beijing, China) in the temperature range of 25–1000 °C, and the average value of the specific heat capacity C_p_ (J/(g·K) was obtained. Samples used in the test were powder with particle size < 187.5 μm.

Thermal conductivity was calculated by Formula (3) [50]. Thermal diffusivity, denoted as α, was measured using a flash thermal conductivity analyzer (NETZSCH LFA 467HT, NETZSCH, Selb, Germany). Five temperature points were selected as 200 °C, 400 °C, 600 °C, 800 °C and 1000 °C and at each temperature point, 3 times test was conducted. The total 15 data was averaged as the thermal diffusivity of the sample. The volume density, denoted as ρ, was measured using an Archimedes densitometer (Model WKT-300C, VicoMeter, Taizhou, China) based on Archimedes’ principle. Samples used for the thermal diffusivity test were round thin sheets with a radius of 1 cm, and samples used for the volume density test were cuboid blocks of 50 mm × 7 mm × 6 mm.

Thermal storage density was calculated by Formula (4) [25] and represents the heat capacity of every unit volume. T_s_ is taken at 25 °C, and T_e_ is taken at 1000 °C in this research. The values were close to other research for better comparison of the thermal storage capabilities of materials.

(1) $$\text{Flexural strength:}\;R=\frac{3\times F\times L}{2\times b\times h^{2}}$$

(2) $$\text{Water absorption:}\;W=\frac{m_{1}-m_{0}}{m_{0}}$$

(3) $$\text{Thermal conductivity:}\;\lambda =\rho \times C_{p}\times \alpha$$

(4) $$\text{Thermal storage density:}\;U=\int_{T_{s}}^{T_{e}}C_{p}\times dT$$

R: flexural strength (MPa), F: maximum load at fracture (N), L: span (mm), b: width (mm), h: minimum thickness at fracture (mm).

W: water absorption (%), m_1_: mass after soaking (g), m_0_: mass before soaking (g).

λ: thermal conductivity (W/(m·K)), ρ: volume density (g/cm^3^), C_p_: specific heat capacity (J/(g·K)), α: thermal diffusivity (mm^2^/s).

U: thermal storage density (J/g), C_p_: specific heat capacity (J/(g·K)), T_s_: the starting temperature of heat storage medium (K), T_e_: the final temperature of heat storage medium (K).

In this study, we conducted an uncertainty analysis to evaluate the reliability of our recorded measurements with the following steps:

A. Identification of Uncertainty Sources: we identified potential sources of uncertainty, including instrument calibration errors, human factors and environmental factors during measurement.

B. Quantification of Uncertainties: Each source of uncertainty was quantified using appropriate statistical methods. Instrumental uncertainties were determined based on the manufacturer specifications, while environmental factors were assessed through repeated measurements under varying conditions.

C. Reporting Uncertainty: The results were reported with their associated uncertainties, allowing for a clear understanding of the reliability and precision of the measurements.

## 3. Results

### 3.1. XRD Result

**Figure 1:**
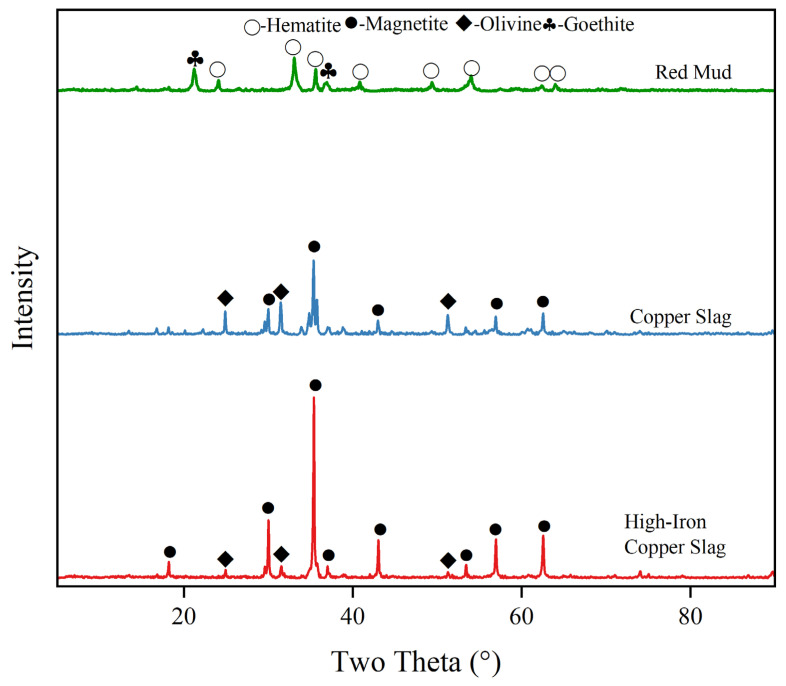
XRD of raw materials.

XRD analysis results of RM, CS and HCS are shown in Figure 1, and it can be found in Figure 1 that the main crystalline phase of RM is hematite, which is consistent with its high iron content in Table 2. The crystalline phase of CS is mainly magnetite and olivine, while the crystalline phase of HCS is mainly magnetite, which is also consistent with the fact that the iron content of HCS is higher, with its silicon content lower compared with those of CS in Table 2.

HCS and RM have similar high iron oxide contents, but the existence form of Fe is quite different. The crystalline phase of HCS is mainly iron olivine and magnetite, while that of RM is mainly hematite and goethite, which is consistent with the silicon content of RM being much lower than HCS.

**Figure 2:**
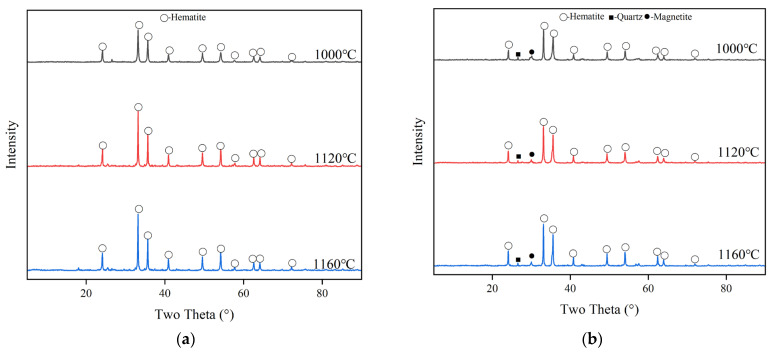

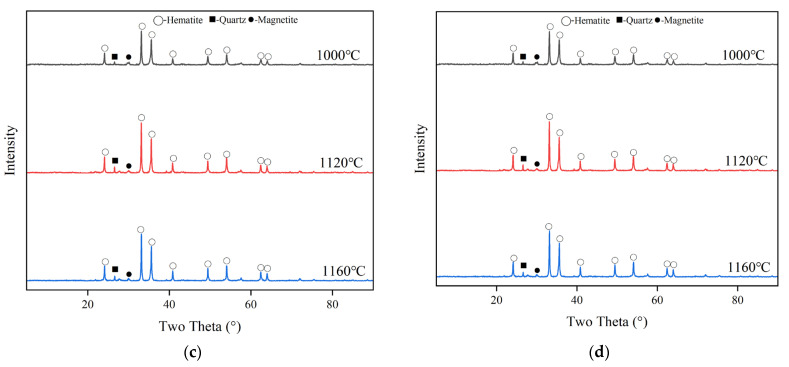
XRD of different groups of TSCs: (**a**) C-RM, (**b**) C-HCS, (**c**) C-CS and (**d**) C-MIX.

In order to clarify the reactions and mineral phase transformations at different temperatures, XRD analyses were carried out on samples sintered at 1000 °C, 1120 °C and 1160 °C of each group, respectively. The aim was to characterize the mineral phase composition of the materials before, during and after sintering, as shown in Figure 2 below.

XRD analysis of C-RM is shown in Figure 2a. The main phase of C-RM is hematite. Likewise, it is shown in Figure 2b that the main phase of C-HCS is hematite with a small amount of magnetite and quartz. As shown in Figure 2c, it can be found that at 1000 °C, 1120 °C and 1160 °C, the main crystalline phase of C-CS is hematite and magnetite. The phase of C-MIX shown in Figure 2d is mainly hematite, quartz and magnetite.

As shown in Figure 2, in the sintering process the diffraction peak of goethite in RM would disappear and convert into hematite. The magnetite phases in both CS and HCS disappear and are oxidized to hematite, with the decomposition of olivine into quartz and magnetite.

### 3.2. Thermal Properties

**Figure 3:**
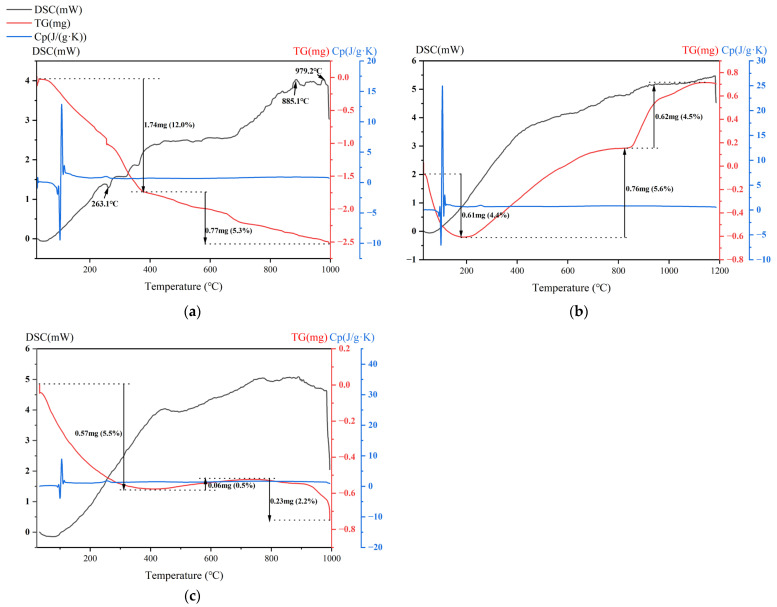
TG-DSC curves of raw materials: (**a**) red mud, (**b**) high-iron copper slag, and (**c**) copper slag (mass changes were marked with black texts; DSC, TG and C_p_ curves were in black, red and blue, respectively).

As can be seen from Figure 3, at about 263.1 °C, RM shows a valley value, which indicates that it undergoes an endothermic reaction at this temperature, which may reveal the removal of crystalline water in the material. At about 885.1 °C and 979.2 °C, the DSC curve shows peaks, which may be related to the exothermic reaction of the material’s solidification. The TG curve of RM shows a downward trend, with a faster decrease in the early stage of 12.0% weight loss and a slower decrease in the later stage of 5.3% weight loss. In the first stage, the weight loss was due to the release of free and bound water during pyrolysis with some carbonate decomposition [51], and the second stage was attributed to the decomposition of FeO(OH) and AlO(OH) of the red mud [52]. The TG curve of HCS shows a downward trend in the early stage and begins to rise at about 182.9 °C. Before 182.9 °C, the 4.4% weight loss was because of free and bound water release, and after that, the mass change was due to phase changes and oxidations of magnetite and olivine [53]. The DSC curve also shows an overall upward trend. The DSC curve of CS shows an overall upward trend, while the TG curve shows a trend of rapid decrease, then a slow increase, and then a rapid decrease. The weight loss in the early stage was caused by the dehydration of moisture and crystal water [54]. The slight mass increase and the weight loss after that were the result of olivine and magnetite transformation and oxidation of sulfide [55]. At around 100 °C, C_p_ values had fluctuations, which was caused by the dehydration reaction of moisture. By calculation, the average specific heat capacity of RM from 25 °C to 1000 °C is 0.72 J/(g·K), that of HCS is 0.76 J/(g·K), and that of CS is 1.26 J/(g·K).

According to the different mechanical properties, samples with excellent properties were selected for thermogravimetric tests, and each of their average specific heat capacity was calculated.

**Figure 4:**
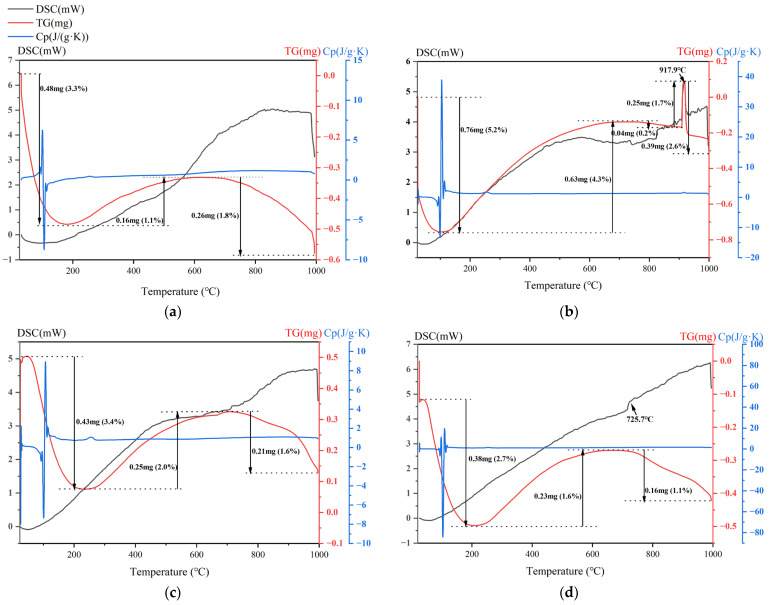
TG-DSC curves of selected TSC samples: (**a**) C-RM, (**b**) C-HCS, (**c**) C-CS and (**d**) C-MIX (mass changes were marked with black texts; DSC, TG and C_p_ curves were in black, red and blue, respectively).

It can be seen from Figure 4a that the DSC curve of sample C-RM5 shows a general upward trend, and the TG curve shows a downward–upward–downward trend. The average specific heat capacity of the sample from 25 °C to 1000 °C is calculated to be 0.67 J/(g·K).

As shown in Figure 4b the DSC curve of sample C-HCS4 rises generally, and the TG curve has a peak at 917.9 °C. The average specific heat capacity of the material is excellent, from 25 °C to 1000 °C, calculated to be 1.27 J/(g·K).

The TG-DSC curve of C-CS5 is shown in Figure 4c. The TG curve of the sample shows a downward–upward–downward trend and the DSC curve shows an upward trend generally. The average specific heat capacity of the material from 25 °C to 1000 °C is 0.80 J/(g·K).

The TG-DSC curve of C-MIX5 is shown in Figure 4d. As can be seen from the figure, the DSC curve of the sample shows an upward trend, and the TG curve shows a downward–upward–downward trend. The average specific heat capacity of the material from 25 °C to 1000 °C is 0.82 J/(g·K).

**Figure 5:**
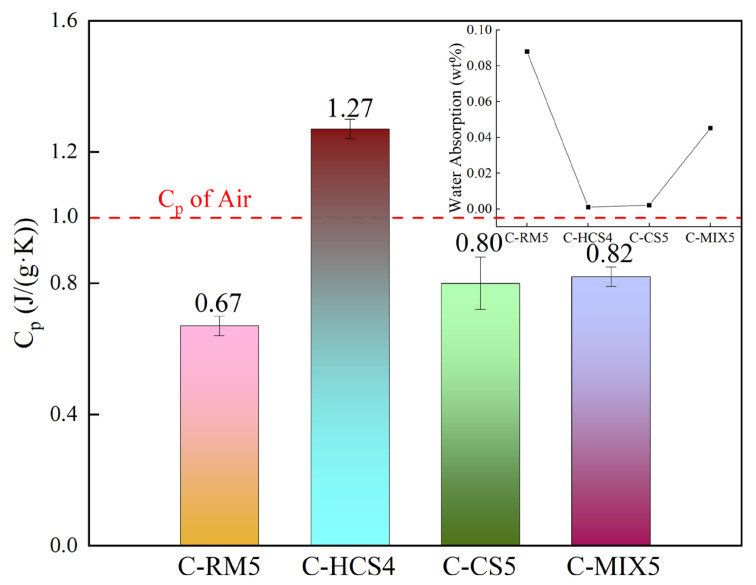
Specific heat capacity of TSC samples.

Figure 5 shows the specific heat capacity of the samples. It also presents a comparison of water absorption of different samples, and it is found that C-HCS4 has the highest Cp and lowest water absorption. The red horizontal line marks the heat capacity of air.

**Figure 6:**
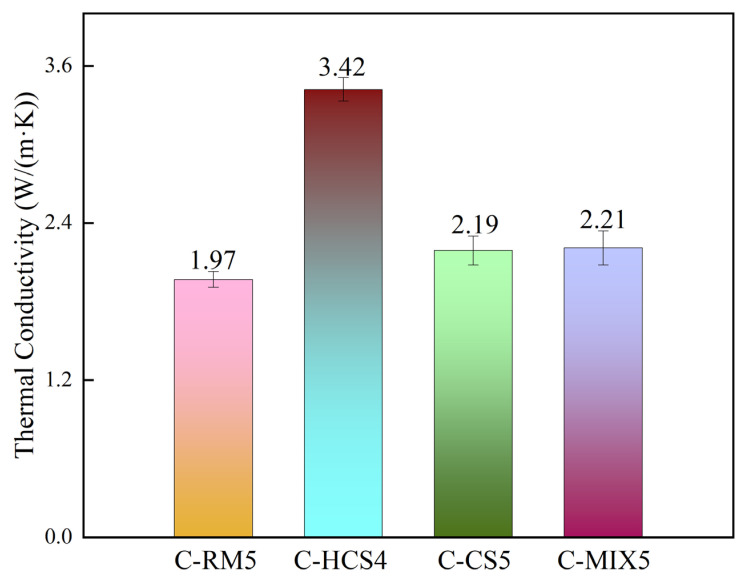
Thermal conductivity of TSC samples.

It can be seen in Figure 6 that the C-HCS4 sample has the highest thermal conductivity of 3.424 W/(m·K), while the C-CS5 and C-MIX5 samples have similar conductivity, and the C-RM5 sample has the lowest.

From above, it is found that the C-HCS4 sample has both the highest specific heat capacity and thermal conductivity of 1.27 J/(g·K) and 3.42 W/(m·K), respectively, and C-RM5 has both the lowest.

### 3.3. Mechanical Properties

**Figure 7:**
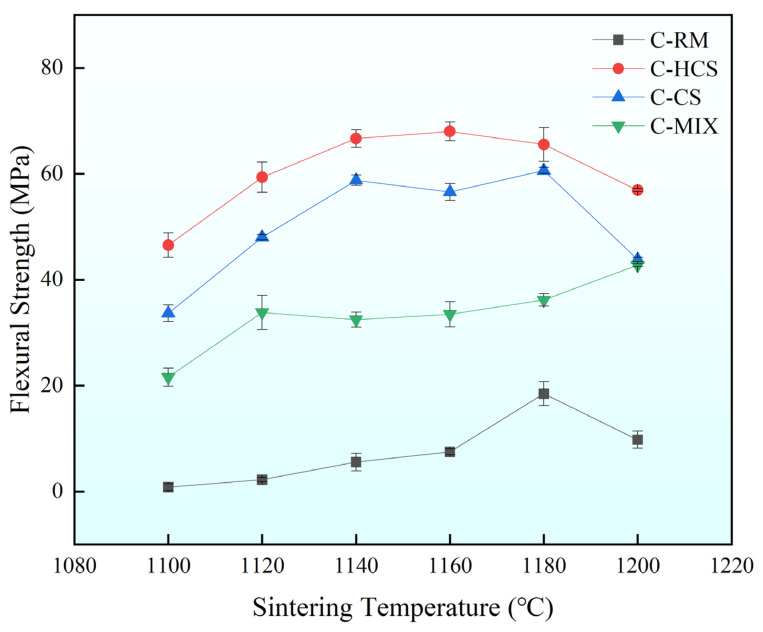
Flexural strength of TSCs under different temperatures.

The flexural strength of each sample was tested and shown in Figure 7 below. It can be found from Figure 7 that the flexural strength of C-HCS is the highest among the four sintered in the range of 1100 °C to 1200 °C, followed by C-CS in second place, C-MIX in third place, and C-RM in last place. Overall, flexural strength shows first an upward trend and then a downward trend as the temperature increases. The flexural strengths of C-HCS can all reach above 40 MPa, and the best performance is 68.02 MPa at 1160 °C. Different from C-HCS, the flexural strength of C-CS is not the highest at 1160 °C, but at 1180 °C, reaching the highest 60.63 MPa. At 1200 °C, the flexural strength of C-MIX reaches the maximum flexural strength of 42.78 MPa and the minimum flexural strength of 21.08 MPa. The flexural strength from C-RM is the lowest, with the highest flexural strength at 18.47 MPa, unable to meet the demands of construction materials.

**Figure 8:**
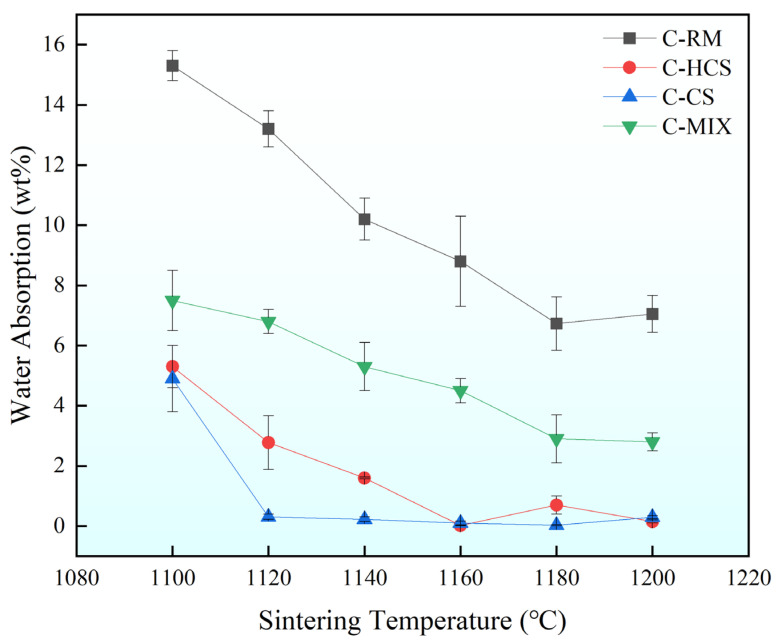
Water absorption of TSCs under different sintering temperatures.

As can be seen from Figure 8, the water absorption of C-CS was the lowest, followed by C-HCS, then C-MIX, and the water absorption of C-RM was the highest. C-CS has a very low water absorption, and it remains relatively stable with temperature above 1120 °C and C-HCS4 has the lowest water absorption. For C-MIX, the water absorption decreases as the temperature increases and reaches 2.8% at 1200 °C. For C-RM samples, the water absorption performances are generally higher than 7%.

By comparing Figure 7 and Figure 8, it can be found that the mechanical properties of the materials sintered at different temperatures generally show an upward–downward trend. Apart from C-RM, flexural strengths of other groups sintered above 1120 °C are relatively high while the water absorption rate first decreases and then increases as temperature increases. The water absorption performances of C-HCS are excellent at 1140 °C, 1160 °C and 1180 °C, and the water absorption performances of C-CS are excellent at 1120–1180 °C.

### 3.4. Microscopic Result

All the samples were tested by SEM-EDS analysis and the results are shown below. In the analysis, elements were selected in EDS analysis according to the element contents.

**Figure 9:**
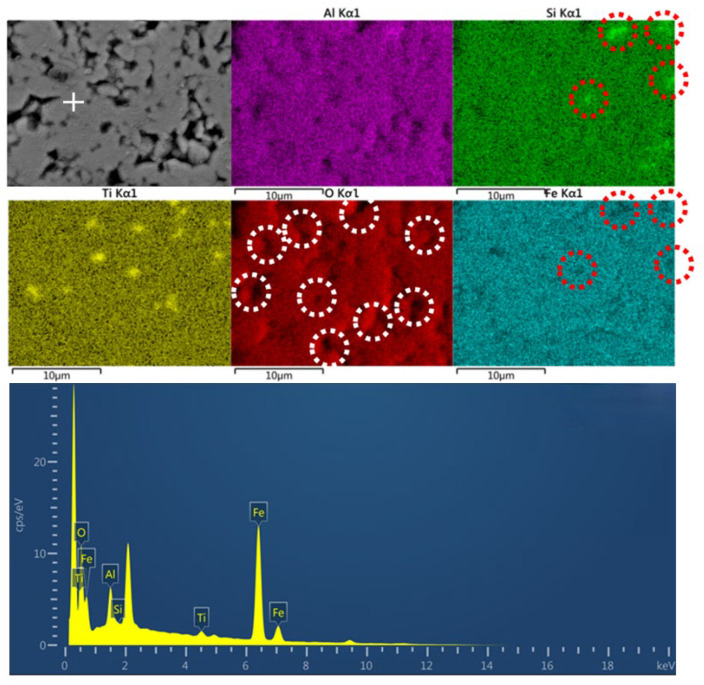
SEM-EDS image of C-RM5.

As shown in Figure 9, there is a certain similarity in the distribution of Al and O elements, and they are mostly concentrated in the light-colored area of the backscattered electron map, forming non-crystalline compounds like silica–alumina. Fe elements are widely distributed but less distributed in the area where Si are concentrated, as red cycles show. Considering Si content in the sample is low and the Fe content is high, it can be inferred that Fe in the sample did not combine with silicon to form the main crystalline phase but mainly formed the hematite phase, which is consistent with the XRD analysis results. It is shown that the hematite grains have small sizes, mostly smaller than 3 μm, and are dispersed in the area in an irregular shape. In addition, the continuous phase concentrates in light-colored areas, which contain hematite phases and aluminum silicate (not detected in Figure 2 but could be deferred [14]) with small grain sizes, and the discontinuous solid phase is mostly composed of some other silicates and quartz phases in the dark-colored areas. Their distribution would exert impacts on thermal conductivity performances. The result of the EDS point scan is shown, and it can be seen in the marked spot that the O element and Fe element have high peaks. Metal coating was conducted in the process to improve the electric conductivity of the material so there is a peak of Pt (the peak without an element label). It was calculated that the atom percentage of O element is 27.38% and the atom percentage of Fe element is 62.34%, so the main crystalline phase is hematite.

**Figure 10:**
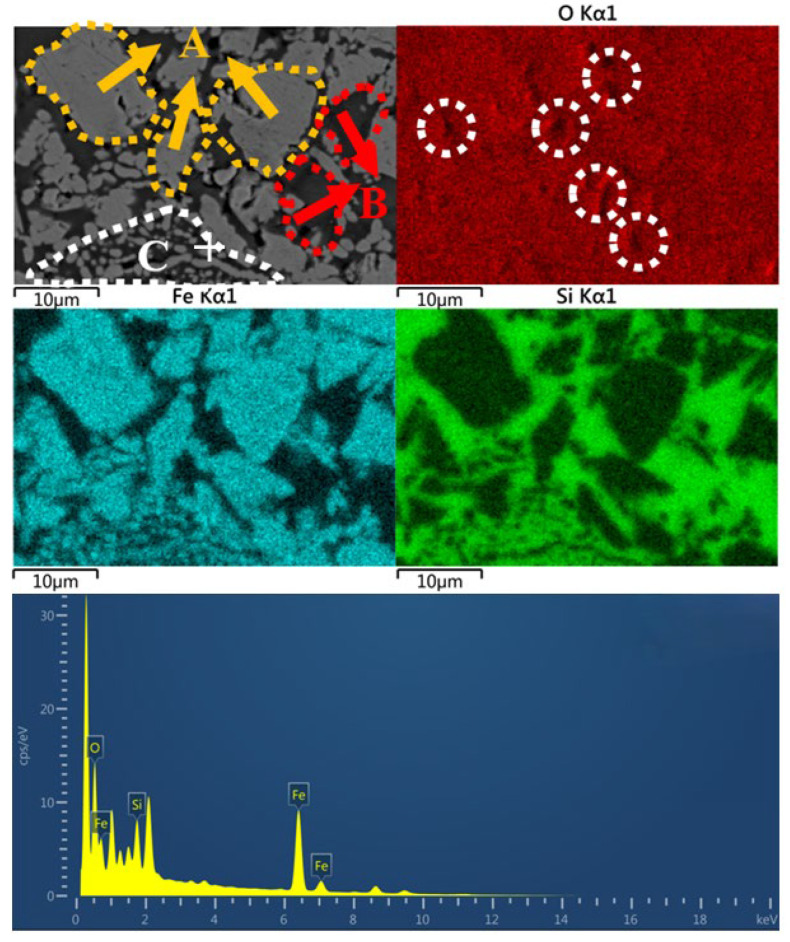
SEM-EDS image of C-HCS4.

As shown in Figure 10, there is a certain correlation between the distribution of Fe and Si elements in the C-HCS4 sample area, which can be divided into three types of regions: A, B and C. Region A is a large, light-colored area with high Fe and O content, mainly composed of iron oxides such as hematite. Region B is a large dark area with high Si and O content, mainly composed of silicon oxides such as quartz. Region C is a light-colored area in granular and strip-like shapes, forming a core-shell structure with Fe and O mostly in the strip-shaped shell and Fe and Si in the core. Grains in region A have large sizes, mostly 10 μm and in block shapes. The continuous phase is concentrated in region A of hematite with large grain sizes and region C of silicates, while the discontinuous solid phase is concentrated in region C of quartz. The EDS point result of the marked spot is shown, and it can be seen that Fe and O have high peaks. The atomic percentages of Fe and O, respectively, are 36.82% and 53.43%, so the atom ratio of Fe and O is close, which means the main phase in this region is magnetite. Metal coating was conducted in the process to improve the electric conductivity of the material so there is a peak of Pt (the peak without an element label).

**Figure 11:**
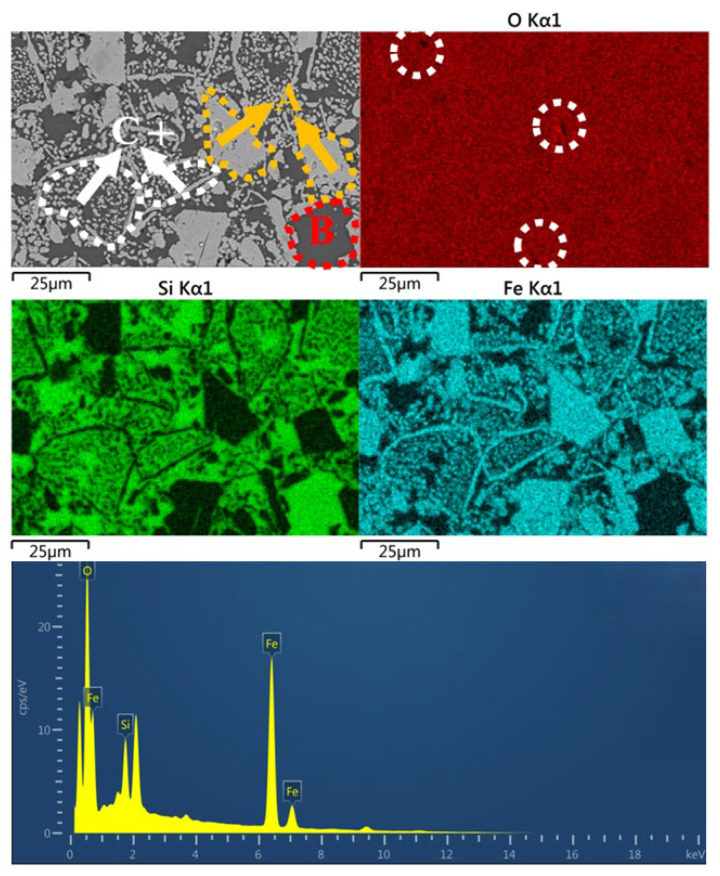
SEM-EDS image of C-CS5.

Figure 11 shows the SEM-EDS image of the C-CS5 sample. From the graph, it can be observed that C-CS5, similar to C-HCS4, also has three regions—A, B and C—and the distribution of the three elements is also similar. Region A is a block-shaped area with grain sizes larger than 25 μm. Similarly, regions A and C are mainly composed of the continuous phase, while region B mainly consists of the discontinuous solid phase. The atom percentage of Fe and O is 50.72% and 42.71% at the marked point, so the main phase in this region is magnetite. Metal coating was conducted in the process to improve the electric conductivity of the material so there is a peak of Pt (the peak without an element label).

**Figure 12:**
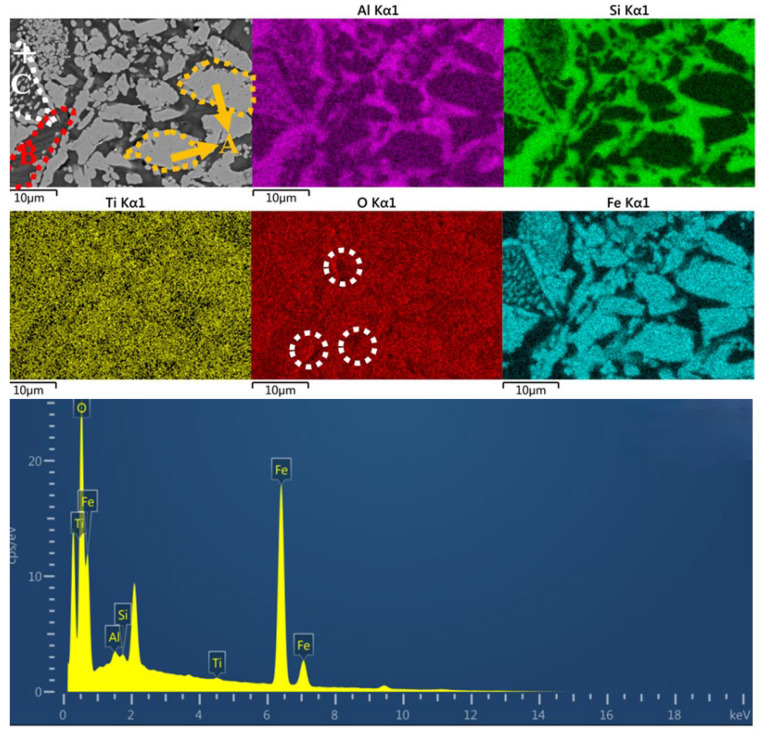
SEM-EDS image of C-MIX5.

Figure 12 shows the SEM-EDS image of the C-MIX5 sample. From the graph, the distribution of Al and Si is similar, mainly distributed in the dark areas, Fe elements are mainly distributed in the block-shaped light areas, and Ti and O are dispersed throughout nearly the whole region. Similar to Figure 10 and Figure 11, this region can also be divided into three regions: A, B and C, with similar element distribution. The sizes of grains in region A are mostly larger than 10 μm and in block shapes. Different from samples C-HCS4 and C-CS5, in the C-MIX5 sample, Al has similar distribution with Si, which means silica–alumina is formed, and in region C, iron oxide and silica–alumina in the inner core have interlaced distribution with hematite as the external shell. The continuous phase mainly concentrates mostly in regions A and C, while the discontinuous solid phase is in region B. The atom percentages of Fe and O are 51.91% and 45.87% at the marked point, while other elements like Ti, Al and Si have low atom percentages. The atom ratio of Fe and O is close to 1, which means the main phase in this spot is magnetite. Metal coating was conducted in the process to improve the electric conductivity of the material so there is a peak of Pt (the peak without an element label).

## 4. Discussion

### 4.1. The Level of Thermal Storage Properties

The properties of samples in this study were compared with other thermal storage materials as Table 4 shows below. As can be seen, the C-HCS4 sample has good thermal storage performances. The specific heat capacity is higher than those of both EAF Slag ceramic and Magnesia Fire Bricks and is nearly two times those of Steel Slag, Corundum and SiC. Thermal conductivity and volume density of C-HCS4 are also higher and thermal storage density is excellent.

### 4.2. Relationship Between Properties and Mineral Phases of TSCs

According to Figure 1, different initial mineral phases of the three raw materials will have a certain impact. When the iron content is similar, Fe of HCS mainly exists in the form of divalent iron ions of magnetite, while that of RM mainly exists in the form of trivalent iron ions of hematite. Although the final mineral phase obtained after sintering is mainly hematite, the reactions during the process are not the same. Both CS and HCS are mainly magnetite, but the difference in iron content results in different material properties. Comparing their performances in Figure 5, Figure 6, Figure 7 and Figure 8 and the results in Table 5, it can be inferred that in case of similar iron content, the properties of materials obtained from divalent iron ions will be superior to those obtained from trivalent iron ions, while when the valence state of iron ions is the same, using raw materials with high iron content can improve the mechanical and thermal properties of the materials. In addition, the mechanical properties of C-MIX are not competitive with C-HCS, which implies the addition of RM into HCS cannot guarantee property improvements.

From the analysis in SEM-EDS, as presented in Figure 9, Figure 10, Figure 11 and Figure 12, it is found that both C-HCS4, C-CS5 and C-MIX5 have magnetite as main phases in region A, so the hematite peaks in XRD analysis of Figure 2 are thought to be overlapping peaks of both hematite and magnetite. The transformation of magnetite to hematite is conducive to the improvement of ceramic properties.

### 4.3. Relationships Between Properties and Microstructure of TSCs

As shown in Figure 9, Figure 10, Figure 11 and Figure 12, C-HCS4, C-CS5 and C-MIX5 samples all show obvious boundary areas of regions A, B and C. However, there are no similar regular areas in C-RM5 samples (shown in Figure 9), and the combination of Fe and Si is not concentrated in the circled area, which means there is less iron silicate formation. Hematite in RM will not go through oxidation reactions in the sintering process, so the mineral phase of C-RM5 will remain in the hematite phase and is dispersed in the area. The high content of Fe_2_O_3_ is conducive to the formation of the liquid phase, which could improve the densification of the material, but meanwhile, the low content of silicate will lead to less amorphous phase formation, which will contribute to the irregular distribution of hematite. It is inferred that this is the reason why the mechanical properties of C-RM5 samples are much lower than those of other samples, as presented in Figure 7 and Figure 8. Although C-RM5 has the highest volume density, its Al in Region B will combine with Si and form silica–alumina, which has a lower heat storage capacity than silica [59]. It can be speculated that the formation of iron oxide and silica in the shape of regions A, B and C is conducive to improving the mechanical and thermal properties.

C-HCS4, C-CS5 and C-MIX5 samples have three kinds of phase regions, as shown in Figure 10, Figure 11 and Figure 12. Region A is a block-shaped area with Fe and O concentrated and is mainly hematite, according to the XRD analysis in Figure 2. It could be inferred that magnetite in CS and HCS is oxidized in the air atmosphere during sintering into hematite. Region B is an amorphous area and is mainly silica or silica–alumina. Region C is a core-shell structure formed from the oxidation of olivine, and in this process, olivine will decompose into hematite and silicate, with the inner core dominated by silica or silica–alumina and hematite, and the outer shell enriched by hematite. Compared with the C-HCS4 sample, C-CS5 has less excellent performance. It is inferred that grain sizes of the main crystalline phase (region A) play a lead role. The C-HCS4 sample has smaller grain sizes than C-CS5, which is the main reason why the C-HCS4 sample has higher flexural strength than the C-CS5 sample.

### 4.4. Relationships Between Properties and Densification of TSCs

Both thermal storage performance and heat conduction performance are greatly affected by the densification of the material, especially the fraction of pores, discontinuous solid phase and continuous phase. At high temperatures, the liquid phase fills in the gaps between solid particles, reducing the apparent pores and forming a continuous phase, while other solid phases are considered discontinuous phases embedded in the continuous phase [47]. If the continuous phases are phases with good thermal properties like iron-rich phases, the thermal performances would be enhanced. In the distribution map of the O element, as shown in Figure 9, Figure 10, Figure 11 and Figure 12, the black part in the figure is marked with a white circle. These parts indicate that the O content in this area is low, and it is speculated to be the porous area of the material. Its distribution not only affects the density, but also thermal storage and heat transfer performance as the air in the pores has different properties from the material, which will directly affect the specific heat capacity and thermal conductivity [60].

The specific heat capacity of C-MIX5 increases most significantly after sintering. The decomposition of some olivine may release free SiO_2_ molecules to form a high-temperature liquid phase, which can improve the transfer of ions and fill pores, thereby promoting the transformation of crystalline phases and the bonding of solid phases, improving the heat storage capacity. Compared with HCS, CS has a lower iron content and a higher silicon content, as shown in Table 2. The chemical compositions of raw materials (wt%) and their initial mineral phase containing more olivine phase are shown in Figure 1. The low iron–silicon ratio is not conducive to the precipitation of crystals in the ceramic, thus decreasing the density. Excessive SiO_2_ will inhibit crystallization and produce amorphous phases after forming a liquid phase at high temperatures [45], resulting in a decrease in the heat storage performance of the material.

Compared with C-CS5, the C-HCS4 sample has a higher content of Fe_2_O_3_, as shown in Table 2, which could improve the formation of the liquid phase and enhance the phase transition efficiency. The higher content of magnetite in the initial phase of C-HCS4 could greatly reduce the melting point and contribute to the liquid phase formation, thus increasing its thermal storage capacity [43,45]. The formation of iron oxide is conducive to the formation of liquid phases penetrating into the pores [61], which increases the densification of the material and thus improves its heat storage capacity. Compared with the C-HCS4 sample, the C-MIX5 sample will form irregular silica–alumina compounds due to the high aluminum content in the added RM, and they are mostly distributed in regions B and C, as shown in Figure 12. Compared with silica, the thermal performance of silica–alumina compounds is not good, which leads to a deterioration of the material’s thermal properties [59,62]. It can be seen from Figure 5 that performance and densification have certain relationships, as water absorption seems inversely proportional to heat capacity. Some studies have already confirmed the opposite relationship between density and thermal conductivity [63]. The main factor impacting the thermal conductivity of an inorganic material is phonon conduction, which is related to the mean free path [27]. The C-RM5 sample is prepared from RM, which contains many impurities, while C-CS5 and C-MIX5 samples have large porosity. Both impurities and pores are material defects, which would greatly reduce the mean free path, thus reducing the thermal conductivity of the material. Based on the analysis above, adding copper slag into red mud could be conducive to the property improvements of the TSC prepared from red mud.

Based on the discussions above, the mineral phases of magnetite and hematite enhancement and grain sizes are key to TSCs’ performances and could be the aim of further research. In addition, attention could also be given to practical applications and communications with thermal storage material companies on the testing of high-temperature electrical conductivity and high-temperature resistance under electric heating conditions. The proposed material is suitable for applications where the material requirements are not high and have large quantity demands.

## 5. Conclusions

Thermal storage ceramics were successfully prepared using red mud, copper slag and high iron copper slag as main materials. Relationships among phase composition, microstructure and the properties of the material were discussed. The conclusions are as follows:

Thermal storage ceramics prepared from high iron copper slag, copper slag and a mixture of red mud and high iron copper slag have good performances. The C-HCS4 sample has excellent properties with a flexural strength of 68.02 MPa, an average specific heat capacity from 25 °C to 1000 °C of 1.27 J/(g·K), and heat conductivity of 3.42 W/(m·K), better than many traditional thermal storage materials. Samples prepared from copper slag and mixture also meet the industrial standard (JC-T 2135-2012) [64], indicating the possibility of heat storage ceramic preparations from these iron-containing solid wastes. All the samples have high iron contents and hematite phases as their main mineral phases, which are conducive to the mechanical and thermal properties.

The hematite phases of these samples have different sources, which contribute to different grain sizes and thus exert impacts on the properties. Hematite in red mud would not go through reactions during sintering, remaining in the dispersed phase with small grain sizes. Magnetite and olivine in copper slag would be oxidized into hematite with large sizes and form a core-shell structure. The different grain sizes would impact the material’s performance.

The hematite in red mud would be stable and influence the densification process, worsening the mechanical and thermal performances of the material. The addition of copper slag into red mud could increase the properties of the thermal storage ceramics prepared from red mud.

The proposed method could reduce the thermal storage material preparation cost because of the cheap and large quantities of raw materials and the concise preparation procedure. The material is particularly suitable in applications where the material requirements are not high and have large quantity demand, like industrial waste heat recovery and solar energy storage systems.

Based on the properties of the materials, it is feasible to use iron-containing bulk solid wastes like red mud and copper slag as raw materials for the low-cost preparation of thermal storage ceramics and achieve the large-scale utilization of these bulk solid wastes.

## Figures and Tables

**Table 1:** Details of thermal storage materials discussed above.

Raw Materials	Preparation Method	Temperature (°C)	Heat Capacity (J/(g·K))	Ref.
Red mud, carbide slag and high-Al cement	Dissolving, stirring, drying, grinding and burning	850	N.A.	[41]
Laterite ore tailing	Leaching, filtrating and roasting	1350–1500	0.79	[42]
Bauxite tailing, red mud and magnetite	Batching, mixing, forming, drying and sintering	950–1100	1.48	[43]
Red mud and shale	Batching, ball milling, sieving, granulation, molding and sintering	980–1140	0.74	[44]
Bauxite tailing, lepidolite and Fe_2_O_3_ powder	Batching, ball milling, drying, granulation, molding and sintering	900–1050	1.06	[45]
Corundum, Fe_2_O_3_ and TiO_2_	Batching, ball milling, pelleting, molding, drying and sintering	1520–1600	1.22	[46]
Fly ash, red mud, clay shale, feldspar and quartz	Batching, grinding, ball milling, pelleting, molding, drying and sintering	1120–1200	1.85	[47]

**Table 2:** Chemical compositions of raw materials (wt%).

	HCS	CS	RM
Fe_2_O_3_	66.79	42.83	67.32
SiO_2_	15.44	35.31	2.71
Al_2_O_3_	4.92	5.16	13.27
ZnO	3.11	3.31	-
CaO	2.27	4.81	0.57
Na_2_O	1.31	1.59	1.42
MgO	1.29	2.57	-
K_2_O	0.96	0.87	-
TiO_2_	0.73	-	3.33
Cr_2_O_3_	0.22	0.09	0.14
MnO	0.25	0.49	0.05
PbO	0.34	0.79	-
CuO	0.29	0.25	-
Co_3_O_4_	0.12	0.11	0.03
MoO_3_	0.33	0.18	-
LOI (Loss On Ignition)	-	-	10.20
Others	1.63	1.64	0.96

**Table 3:** Contents of raw materials in different TSC samples (wt.%).

TSCs	HCS	CS	RM	Fe_2_O_3_	SiO_2_	Al_2_O_3_	CaO
C-RM	0	0	100	67.32	2.71	13.27	0.57
C-HCS	100	0	0	66.79	15.44	4.92	2.27
C-CS	0	100	0	42.83	35.31	5.16	4.81
C-MIX	80	0	20	66.89	12.89	6.59	1.93

**Table 4:** Properties of Samples.

Materials	Specific Heat Capacity (J/(g·K))	Thermal Conductivity (W/(m·K))	Volume Density (g/cm^3^)	Thermal Storage Density (J/g)	Thermal Storage Density (kWh/m^3^)	Ref.
C-HCS4	1.27	3.42	4.12	1238.25 (25–1000 °C)	1417.11 (25–1000 °C)	This Study
EAF Slag Ceramic	1.00	1.10	3.08	900 (100–1000 °C)	770 (100–1000 °C)	[56]
Magnesia Fire Bricks	1.15	1.50	3.00	1345.5 (30–1200 °C)	1121.25 (30–1200 °C)	[21]
Steel Slag	0.66	N.A.	3.03	867.9 (25–800 °C)	730.48 (25–800 °C)	[27]
Corundum	0.70	N.A.	N.A.	837 (25–800 °C)	N.A.	[57]
SiC	0.68	N.A.	N.A.	N.A.	N.A.	[58]

**Table 5:** Performances and mineral phase of samples.

	Flexural Strength (MPa)	Volume Density (g/cm^3^)	Specific Heat Capacity (J/(g·K)) (25–1000 °C)	Thermal Conductivity (W/(m·K)) (25–1000 °C)	Main Mineral Phase
C-RM5	18.47	4.25	0.67	1.97	Hematite
C-HCS4	68.02	4.12	1.27	3.42	Hematite, magnetite, quartz
C-CS5	60.63	3.76	0.80	2.19	Hematite, magnetite, quartz
C-MIX5	36.19	3.56	0.82	2.22	Hematite, magnetite, quartz

## Data Availability

The original contributions presented in the study are included in the article; further inquiries can be directed to the corresponding author.
